# Co-Exposure with an Invasive Seaweed Exudate Increases Toxicity of Polyamide Microplastics in the Marine Mussel *Mytilus galloprovincialis*

**DOI:** 10.3390/toxics10020043

**Published:** 2022-01-18

**Authors:** Filipa G. Rodrigues, Hugo C. Vieira, Diana Campos, Sílvia F. S. Pires, Andreia C. M. Rodrigues, Ana L. P. Silva, Amadeu M. V. M. Soares, Jacinta M. M. Oliveira, Maria D. Bordalo

**Affiliations:** 1 Department of Biology, University of Aveiro, 3810-193 Aveiro, Portugal; filipagrodrigues@ua.pt; 2 CESAM—Centre for Environmental and Marine Studies, Department of Biology, University of Aveiro, 3810-193 Aveiro, Portugal; hugovieira@ua.pt (H.C.V.); silviapires1@ua.pt (S.F.S.P.); rodrigues.a@ua.pt (A.C.M.R.); ana.luisa.silva@ua.pt (A.L.P.S.); asoares@ua.pt (A.M.V.M.S.); jacintaoliveira@ua.pt (J.M.M.O.); maria.bordalo@ua.pt (M.D.B.)

**Keywords:** invasive macroalgae, bivalves, marine debris, oxidative stress, energy balance, byssus production

## Abstract

Plastic pollution and invasive species are recognised as pervasive threats to marine biodiversity. However, despite the extensive on-going research on microplastics’ effects in the biota, knowledge on their combination with additional stressors is still limited. This study investigates the effects of polyamide microplastics (PA-MPs, 1 mg/L), alone and in combination with the toxic exudate from the invasive red seaweed *Asparagopsis armata* (2%), after a 96 h exposure, in the mussel *Mytilus galloprovincialis*. Biochemical responses associated with oxidative stress and damage, neurotoxicity, and energy metabolism were evaluated in different tissues (gills, digestive gland, and muscle). Byssus production and PA-MP accumulation were also assessed. Results demonstrated that PA-MPs accumulated the most in the digestive gland of mussels under PA-MP and exudate co-exposure. Furthermore, the combination of stressors also resulted in oxidative damage at the protein level in the gills as well as in a significant reduction in byssus production. Metabolic capacity increased in both PA-MP treatments, consequently affecting the energy balance in mussels under combined stress. Overall, results show a potential increase of PA-MPs toxicity in the presence of *A. armata* exudate, highlighting the importance of assessing the impact of microplastics in realistic scenarios, specifically in combination with co-occurring stressors, such as invasive species.

## 1. Introduction

Marine environments represent an important life support system and one of the most complex ecosystems [1]. Nevertheless, biodiversity and marine resources are increasingly endangered due to pollution and other anthropogenic issues associated with the fast pace of human population growth and the development of the economy. The introduction of non-native marine species, overfishing, global climate change, and habitat destruction and modification are key pressure points, especially in coastal areas [2].

Global plastic production has increased dramatically in recent years, reaching almost 370 million tonnes in 2019 [3], raising growing scientific and societal concerns. In particular, microplastics (MPs: <5 mm in size) are an emerging environmental issue that accounts for the major percentage of plastic litter, having been detected in many environmental matrices [4]. These polymers are introduced in marine ecosystems through multiple pathways, such as direct disposal, airborne dispersal, terrestrial runoff, and riverine flow [5,6]. MP levels are expected to range between <0.0001 and 1.89 mg/L in the marine environment [7]. However, as these particles undergo continuous fragmentation, and considering that most surveys do not detect particles <300 μm, the concentrations found in the environment are probably underestimated [8]. Several studies have observed that MPs are widely available to the marine food web [9], as they are very similar in size to various organisms in the planktonic and benthic communities [9]. The intake of MPs can occur via gills or through direct consumption (i.e., particle ingestion) or indirectly (i.e., via trophic chains) [6,10]. Therefore, the bioavailability of MPs to marine biota is the primary environmental risk associated with this pollutant [9,11]. In this regard, filter-feeding marine organisms, such as bivalves, are probably among the most impacted groups, since they can involuntarily ingest these synthetic materials along with the natural food items while feeding by constantly filtrating substantial volumes of seawater [12]. Once ingested, small-sized MPs can be taken up into the cells by endocytosis and are accumulated or translocated to different tissues in the organisms [13,14,15]. MP intake may, therefore, lead to histological alterations, inflammatory reactions, and ecotoxicological responses at cellular, molecular, and biochemical levels, as they are responsible for detrimental modulations of biological functions, such as reproduction, growth, survival, and feeding [9,16]. 

There are different types of plastic polymers and one of the most common groups includes polyamides (PA) [17], which are important engineering plastics often used in domestic and automotive industries [18] due to their high durability and resistance. Furthermore, these particles may be released from fishing gear and aquaculture facilities [6,19], and are frequently detected in coastal waters, including biotic [20,21], water [22], and sediment compartments [23]. PA particles can be found from the intertidal to the subtidal environments [24], as they have a density similar to seawater, allowing them to remain suspended in the water-column [10], remaining available as a “food item” for filter-feeding marine organisms.

The proliferation of invasive species has also been a major cause of concern in marine ecosystems, posing a threat to biodiversity and potentially leading to severe alterations in the functioning and structure of the ecosystem. In particular, marine macroalgae constitute the main component of introduced biota, with a current global estimate varying from 163 to over 300 species [25]. The northeast Atlantic and the Mediterranean coasts support the largest number of macroalgae introductions [26], with the main human-mediated vectors responsible for their transport being maritime traffic (e.g., hull fouling, ballast waters), aquaculture, and aquarium trade [27]. Once non-native macroalgae spread beyond their natural distribution through human activities and become successfully established, they are defined as invasive [28], competing with native species, and potentially leading to their displacement. Invasive species may also modify habitats and their structure, promoting biodiversity loss, and creating cascading effects or changes in the food chain [29], which may cause significant ecological and economic damages [30]. *Asparagopsis armata* Harvey, 1855 is a red seaweed native to Southern Australia and New Zealand [31], first described in the Atlantic and Mediterranean coasts in the 1920s [32], as it is widely distributed from the British Isles to Senegal [33,34], including the Azores Islands and mainland Portugal [35,36]. It is globally known for strong invasive behaviour due to its type of life cycle (leading to fast and vast propagation mainly due to its free-living stage) and lack of predators in the invaded habitat [37]. Exudation of secondary metabolites, including halogenated compounds such as haloforms, haloketones, and haloacids, constitutes a chemical defence mechanism that is a key aspect for *A. armata* invasiveness by becoming unpalatable for predators [38,39]. Thus, this seaweed has been considered an important source of bioactive metabolites with antibacterial and antifungal properties [40], and some were also found to have mutagenic and cytotoxic effects [41]. This red macroalga is mainly found from the low intertidal to the shallow subtidal zone [42], often attached to the substrate or drifting, and tend to concentrate in rock pools during low tide [43]. In this type of environment, such chemical compounds, once exuded into the water, may be potentially toxic and pose a threat to native biota [43]. Some previous studies have already devoted attention to the impact of *A. armata* exudate on the surrounding biota. For instance, exposure to *A. armata* halogenated metabolites caused physiological impairment on the crustacean *Palaemon elegans*, the gastropod *Gibbula umbilicalis*, and the mussel *Mytilus galloprovincialis* [43,44,45]. Low exudate concentrations were also found to reduce feeding activity of *G. umbilicalis* and *M. galloprovincialis* as well as the byssal production and strength of *M. galloprovincialis* [44,45]. Moreover, a tendency of an increasingly toxic action of the exudate was observed in *M. galloprovincialis* under a warming temperature scenario [46]. 

Mussels are abundant, widespread bivalves, and key players within marine trophic chains, being frequently selected as sentinel organisms and used in ecotoxicological studies for monitoring coastal environments as representative of low-trophic level organisms [47]. The mussel *M. galloprovincialis* is considered an ecologically important organism in coastal waters and is frequently used as a bioindicator of MP pollution in marine environments [4]. The sedentary and suspension filter-feeding behaviours of this mussel species translates in a great capacity to uptake and accumulate many contaminants, consequently providing a specific response that reflects the effects of different perturbations [48]. Furthermore, this species represents an important link between benthic and pelagic ecosystems [4] and forms dense monolayered and multi-layered beds attached to the hard substrate along intertidal rocky shores providing habitat structures and shelter to various organisms, increasing habitat complexity and enhancing the biodiversity [49]. *M. galloprovincialis* also has a high socio-economic value, representing an important food resource globally consumed by human populations due to its nutritional relevance, hence representing one of the most harvested and produced species, particularly in Portugal [50].

Considerable investigations have been carried out on the effect of different MPs in the mussel, *M. galloprovincialis* [4,12,14,48,51], but none studied the consequence of this exposure in co-occurrence with the exuded compounds from an invasive seaweed. The presence of different stressors in the environment may lead to complex interactions and scenarios that need to be taken into account when evaluating their impact in order to identify realistic scenarios of exposure. Furthermore, despite being a commonly found polymer in coastal waters [20,21,22], there is a knowledge gap of the effect of PA-MPs in marine organisms. In this sense, the present study aimed to evaluate the consequences of PA-MP exposure in the mussel *M. galloprovincialis* and assess the influence of *A. armata* exudate on the impacts caused by this polymer. Physiological responses, including byssal thread production, oxidative damage, antioxidant defences, enzymatic activity for cholinergic neurotransmission, energy production, and metabolism, were measured.

## 2. Materials and Methods

### 2.1. Asparagopsis armata Sampling and Exudate Production 

The red macroalga *A. armata* (gametophyte phase) was collected by hand through free diving in the subtidal zone at the Terceira Island in Azores (Portugal) (38°38′59.2″ N, 27°13′16.4″ W). After collecting, the macroalgae were kept in aerated seawater tanks until the next day and packed in sealed containers to be transported to the laboratory in Aveiro (Portugal). Upon arrival, *A. armata* was immediately cleared from any perceptible associated fauna and debris. Afterwards, they were allocated to a tank with artificial seawater (marine RedSea^®^ Salt premium grade) in a 1:10 proportion (salinity: 35 ± 1, pH: 8.0 ± 0.1, temperature: 20.0 ± 0.5 °C) in the dark and with no aeration for 24 h to produce the exudate, adapted from [45]. Algae were then removed from the tank and the resulting media (considered as the stock solution, representing 100% of exudate) was preserved at −20 °C. When needed, the exudate was slowly defrosted in the dark at 4 °C, and used at a 2% concentration, chosen according to previous sublethal toxicity test results [45].

### 2.2. Mytilus galloprovincialis Sampling and Acclimation

In December 2020, adult specimens of *M. galloprovincialis* (4.2 ± 0.1 cm shell length) were harvested by hand, on the intertidal rocky shore of the Barra of Aveiro in Portugal (40°38′38.8″ N, 8°44′44.6″ W), during low tide. Mussels were measured with a pachymeter in the field and then transported to the laboratory, where the shell surface was gently scraped to remove algae, encrusting organisms, and debris. Afterwards, *M. galloprovincialis* individuals were allowed to depurate and acclimate during seven days in glass aquariums that contained aerated artificial seawater (salinity: 30.0 ± 0.5; temperature: 19.0 ± 0.5 °C; pH: 8.0 ± 0.1; dissolved oxygen: 8.0 ± 0.5 mg/L; oxygen saturation: >80%, measured with WTW portable meters, Weilheim, Germany) in a recirculating aquatic system (a flow-through system ensured continuous seawater renewal), with a 14 h light:10 h dark photoperiod. 

### 2.3. Microplastic Preparation

Polyamide microplastics (PA-MP, mean size: 30–50 μm, irregularly shaped, density: 1.14 g/cm^3^; CAS 32131-17-2, Figure S1) were generously provided by a company that chose to remain anonymous. A stock solution (100 mg PA-MP/L) was prepared in artificial seawater (salinity: 30; RedSea^®^ Salt premium grade mixed with reverse osmosis water) previously filtered (0.45 μm pore size). This PA-MP solution was allowed to equilibrate for 96 h at 50 rpm at room temperature in the dark. A solution containing only artificial seawater to be used in the treatments without PA-MPs was prepared and left to shake in the same conditions. The final concentration was achieved by adding 5 mL of the stock solution to the test vials containing 495 mL of seawater, resulting in a final concentration of 1 mg/L, which fits within realistic environmental MP concentrations [7]. In the treatments without PA-MP, 5 mL of the aged artificial seawater were also added.

### 2.4. Experimental Setup

After acclimation, 48 mussels were exposed for 96 h to the following treatments: (i) control (artificial seawater only); (ii) *A. armata* exudate (2% concentration); (iii) PA-MPs (1 mg/L); and (iv) *A. armata* exudate (2%) and PA-MPs (1 mg/L), simultaneously. The 96 h exposure was selected in accordance with American Society for Testing and Materials E729-96 [52]. For each treatment (control; exudate exposure; PA-MP exposure; and exudate and PA-MP exposure), 12 replicates were used with 1 mussel placed individually in 1 L glass flasks containing 500 mL of aerated test medium (static exposure). Seven replicates were used for the biomarkers’ analysis, and the remaining five replicates were used for PA-MP quantification. The physical–chemical test parameters were maintained at salinity—30.5 ± 0.3, temperature—18.0 ± 0.3 °C, pH—8.0 ± 0.2, dissolved oxygen—8.0 ± 0.5 mg/L, oxygen saturation—>83%, and a 14 h light:10 h dark photoperiod was used. After the 96 h of exposure, the soft tissues of each mussel were removed using a scalpel and tweezers. Tissue samples (gills, muscles, and digestive gland) for the biomarkers analysis were individually stored and weighed in microcentrifuge tubes, frozen in liquid nitrogen and subsequently stored at –80 °C prior to further analysis. Samples for the PA-MP quantification (gills and digestive gland) were kept in small glass flasks (for the microplastic quantification) and preserved at −20 °C.

### 2.5. Digestion of Mussel Tissues and Microplastic Quantification

The digestion and filtration procedures were adapted from the method developed by Prata et al. [53].

A 10% potassium hydroxide (KOH) (*w*/*v* ≥ 85%, Fisher Scientific, Loughborough, UK, CAS 1310-58-3) solution (100 g of KOH pellets dissolved in 1000 mL Milli-Q ultra-pure water) was freshly prepared and used to digest the mussels’ tissues. Ten mL of the KOH solution were added to each glass flask containing the samples, covered with aluminium foil, and incubated at 50 °C for 48 h. After the incubation period was over, the filtration of the samples followed. 

The samples were heated to boiling just before being filtered to improve the solubility of fats and soaps and, consequently, the filtration rates. Then, samples were vacuumed filtered onto glass microfiber filters (47 mm Ø; 1.2 μm pore size, Prat Dumas, Couze-St-Front, France), washed with 50 mL of boiling Milli-Q ultra-pure water, followed by the addition of 10 mL of acetone (99.5+%, Fisher Scientific, Loughborough, UK, CAS 67-64-1). Samples were then incubated for 10 min and washed with ultra-pure water. 

To assure quality control during testing, the glassware was acid-washed and rinsed with Milli-Q ultra-pure water; procedural blanks (1 per every 10 samples) were prepared with the KOH solution and received the same treatment as the other samples; for digestion, tissue samples were prepared and handled under a laminar flow chamber.

After drying, each glass fibre filter of each sample (including blanks) was observed under a stereomicroscope (Zeiss, Stemi 2000, Jena, Germany), and the number of PA-MP particles was visually counted. All fibres were discarded from the analysis. In case of any doubt, PA-MPs were confirmed by applying the method of hot needle [54]. The number of PA-MPs is presented as the number of counted particles/g tissue/organism.

### 2.6. Biomarker Analysis

#### 2.6.1. Sample Preparation

Samples of *M. galloprovincialis* tissues (gills, muscles, and digestive glands) were individually homogenised on ice through sonication (10% pulse mode, 250 Sonifier, Branson Ultrasonics, Danbury, CT, USA) using 1500 μL 0.1 M K-phosphate buffer, pH—7.4. Muscle samples to be analysed for energy metabolism were homogenised using the same procedure in 1500 μL ultra-pure water.

After homogenisation, one aliquot from each gill, digestive, and muscle replicate was stored with 4% butylated hydroxytoluene (BHT) in methanol to evaluate the lipid peroxidation (LPO). Aliquots for protein carbonylation (PC) determination were also stored. The remaining homogenate of gills and digestive samples was centrifuged for 15 min at 10,000 g (4 °C), and the obtained post-mitochondrial supernatant (PMS) was divided into microtubes and kept in −80 °C for posterior analysis of catalase (CAT), glutathione S-transferase (GST), and acetylcholinesterase (AChE) activities, and total glutathione (tGSH) content. The PMS from the muscle homogenate was used for determining AChE activity in this tissue.

Aliquots of muscle homogenates were also stored for the analysis of lactate dehydrogenase (LDH) activity, proteins, lipids, and sugars contents, and electron transport system (ETS) activity.

Biomarkers determinations were done in micro-assays set up in 96-well flat bottom plates and read spectrophotometrically (Microplate reader MultiSkan Spectrum (Thermo Fisher Scientific, Waltham, MA, USA).

#### 2.6.2. Oxidative Stress and Neurophysiological Biomarkers

The protein concentration of PMS was determined according to the Bradford method [55], using bovine-globulin as a standard. The Ellman’s method [56], adapted to the microplate [57], was applied to measure acetylcholinesterase (AChE) activity, using acetylthiocholine as substrate and following the absorbance increase at 412 nm. Catalase (CAT) activity was measured in the PMS by following the decomposition of the substrate hydrogen peroxide (H_2_O_2_) at 240 nm [58]. Glutathione-*S*-transferase (GST) activity was measured in PMS after the conjugation of reduced glutathione (GSH) with 1-chloro-2,4-dinitrobenzene (CDNB) at 340 nm [59]. The total glutathione (tGSH) content was determined in the PMS fraction using the recycling reaction of GSH with 5,50-dithiobis-(2-nitrobenzoic acid) (DTNB) in the presence of glutathione reductase (GR) excess at 412 nm [60,61,62]. To determine endogenous lipid peroxidation (LPO) thiobarbituric acid-reactive substances (TBARS) were measured at 535 nm [63]. Protein carbonylation (PC) was quantified at 450 nm based in the reaction of 2,4-dinitrophenylhydrazine (DNPH) with carbonyl groups, according to the DNPH alkaline method [64]. Lactate dehydrogenase (LDH) activity was determined by following the NADH oxidation caused by pyruvate consumption, as it leads to the decrease of absorbance at 340 nm [65], adapted to the microplate [66].

#### 2.6.3. Cellular Energy Allocation (CEA)

CEA value is obtained from the ratio between Ea, the energy available (the sum of proteins, lipids, and sugar contents), and Ec, which is aerobic energy production (estimation of ETS activity). The CEA and ETS activity were determined based on the methods described by De Coen and Janssen [67], slightly modified for the microplate [68].

Total lipid content in muscle tissue was determined by adding chloroform, methanol, and ultra-pure water in a 2:2:1 proportion. In the organic phase of each sample, sulfuric acid (H_2_SO_4_) was added, followed by an incubation period of 15 min at 200 °C, and the absorbance was measured at 375 nm using tripalmitin as a lipid standard. To determine the carbohydrate and protein contents, 15% thiobarbituric acid (TCA) was added to 300 μL of homogenate and incubated for 10 min at −20 °C. Carbohydrate quantification was performed in the supernatant by adding 5% phenol and H_2_SO_4_ to the samples, and the absorbance was read at 492 nm, using glucose as a standard. For total protein content quantification, the remaining pellet was resuspended with 1 M NaOH (incubated for 30 min at 60 °C) and then neutralized with 1.67 HCl. Total protein content quantification followed the Bradford’s method [55], using bovine serum albumin as a standard and measuring absorbance at 520 nm. Proteins, lipids, and sugar fractions were converted into energetic equivalent values using the corresponding energy of combustion: 24,000 mJ/g, 39,500 mJ/g, and 17,500 mJ/g, respectively [69]. 

Electron transport system (ETS) activity was evaluated using the INT (Iodonitrotetrazolium chloride) reduction assay by measuring the rate of INT reduction in the presence of the non-ionic detergent Triton X-100, at 490 nm. The stoichiometric relationship in which for 2 μmol of formazan formed, 1 μmol of oxygen is consumed was applied to calculate the cellular oxygen consumption rate. The final Ec value was converted into an energy equivalent using the specific oxyenthalpic equivalent for an average lipid, protein, and carbohydrate mixture of 480 kJ/mol O_2_ [69].

### 2.7. Byssal Thread Production

The quantity of produced byssal threads was assessed as a physiological biomarker. Once the 96 h exposure period for the different treatments (0% exudate; 2% exudate; PA-MPs; and 2% exudate and PA-MPs) ended, the number of functional byssus produced by each *M. galloprovincialis* individual was counted, according to Coelho et al. [45]. For this evaluation, all 12 replicates were used.

### 2.8. Statistical Analysis

The statistical analysis of data and graphical representations of results was performed using IBM SPSS Statistics 27 and GraphPad Prism 9 for Windows. Data normality and homoscedasticity were assessed on the residuals, using the Shapiro–Wilk Test (*p* > 0.05) and the Levene’s Test (*p* > 0.05), respectively. For variables not showing a normal distribution or homoscedasticity, data were square root (CAT, GST, LPO, AChE, tGSH, AChE, LDH, and AChE in the muscle) or log-transformed (lipid content, ETS activity, Ea, and PA-MP quantification in the digestive gland). 

Parametric t-tests were performed to evaluate differences in the number of PA-MP particles per tissue between treatments exposed to PA-MP. One-way analysis of variance (ANOVA) with a post hoc Dunnet’s test was used to investigate treatment-dependent effects on byssus production. Effects on biochemical responses among *A. armata* exudate, PA-MPs and their interactions after exposure were evaluated through two-way ANOVA, using *A. armata* exudate and PA-MPs as factors (IBM SPSS Software, Armonk, NY, USA). The *post hoc* Šídák’s test was used to perform multiple comparisons and identify significant differences between treatments (GraphPad Software, CA, USA). Data were presented as mean value (mean) ± standard error of mean value (SEM).

## 3. Results

### 3.1. Polyamide Microplastics Quantification

PA-MP particles were found mainly in the digestive gland and, at a lesser amount, in the gills (Table 1). Despite the observed increase in the number of particles between the PA-MP treatment and the combined exposure, this difference was not significant (*p* > 0.05).

### 3.2. Oxidative Stress and Neurophysiological Biomarkers

**Figure 1:**
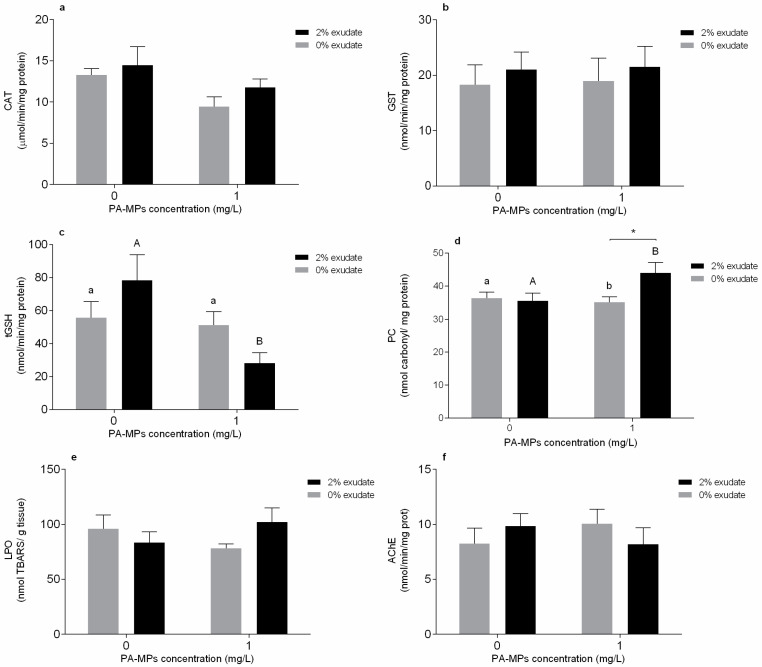
Oxidative stress-related biomarkers of *Mytilus galloprovincialis* gills after 96 h of exposure to *A. armata* exudate (0% and 2%) at different polyamide microplastic (PA-MPs) concentrations (0 and 1 mg/L). (**a**) Catalase activity (CAT), (**b**) glutathione-*S*-transferase activity (GST), (**c**) total glutathione contents (tGSH), (**d**) protein carbonylation levels (PC), (**e**) lipid peroxidation (LPO), and (**f**) acetylcholinesterase activity (AChE). All values are presented as mean ± SEM. * denotes a significant difference between the 0% and 2% *A. armata* exudate in the same PA-MPs concentration. The upper-case letters indicate differences in the 0% exudate treatments and the different lower-case letters represent differences in the 2% exudate treatments at the different PA-MPs concentrations.

In the gills, a significant effect of PA-MPs factor was observed for CAT activity of exposed mussels (Table S1); however, despite the observed tendency to decrease CAT activity, the *post hoc* test could not discriminate significant differences among the several treatments (Figure 1a). Considering the GST activity (Figure 1b), no significant changes in the presence of *A. armata* exudate, PA-MPs, or even by the interaction between *A. armata* exudate and PA-MPs were observed (Table S1). On the other hand, significant effects were observed in the levels of tGSH in the presence of PA-MPs and in mussels exposed to both stressors, reflected by the significant interaction between *A. armata* exudate and PA-MPs (Table S1). Specifically, there were significant differences within the 2% exudate concentration (*p* < 0.05); i.e., the tGSH levels exhibited a decrease in the mussels exposed to exudate in the presence of PA-MPs, when compared to the single exposure of *A. armata* exudate (Figure 1c).

Regarding the oxidative damage in the mussel gills, no changes in PC levels were observed in mussels exposed to PA-MPs and *A. armata* exudate; however, the interaction between these factors significantly affected PC levels (Table S1). Furthermore, PC levels demonstrated a significant difference in mussels exposed to the 2% exudate concentration (*p* < 0.05), with increased values in the exposure to *A. armata* exudate in the presence of PA-MPs, when compared to the 2% exudate treatment (Figure 1d). A significant difference within the 1 mg PA-MP/L (*p* < 0.05) was also verified, whereas the exposure to PA-MPs in the presence of 2% exudate exhibited superior PC levels when compared with the exposure to PA-MPs without exudate (Figure 1d). On the other hand, LPO was not significantly affected by *A. armata* exudate, PA-MPs, or their interaction (Table S1, Figure 1e). Regarding neurotoxicity, none of the experimental treatments resulted in significant effects (*p* > 0.05) in the AChE activity (Table S1, Figure 1f).

**Figure 2:**
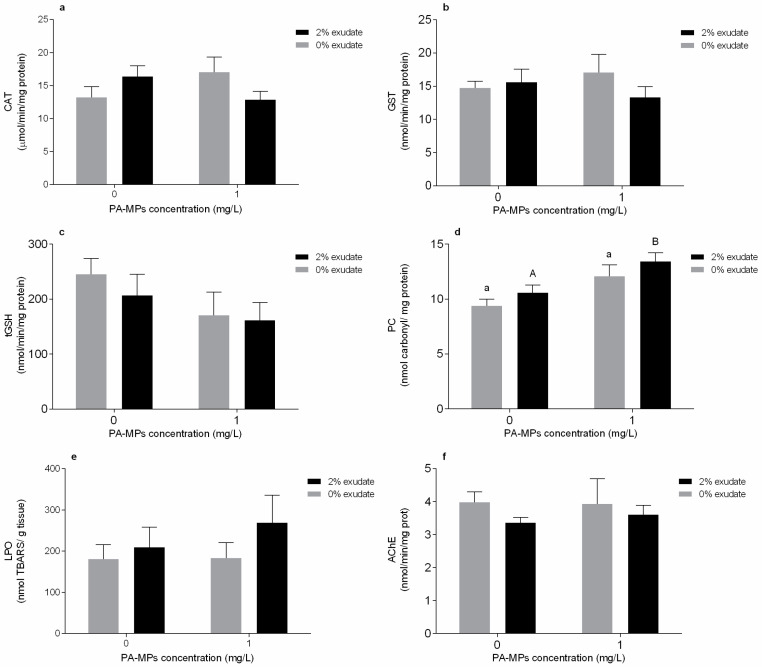
Oxidative stress-related biomarkers of *Mytilus galloprovincialis* digestive gland after 96 h of exposure to *A. armata* exudate (0% and 2%) at different polyamide microplastic (PA-MPs) concentrations (0 and 1 mg/L). (**a**) Catalase activity (CAT), (**b**) glutathione-*S*-transferase activity (GST), (**c**) total glutathione contents (tGSH), (**d**) protein carbonylation levels (PC), (**e**) lipid peroxidation (LPO), and (**f**) acetylcholinesterase activity (AChE). All values are presented as mean ± SEM. The upper-case letters indicate differences in the 0% exudate treatments, and the different lower-case letters represent differences in the 2% exudate treatments at the different PA-MPs concentrations.

In the digestive gland, no significant effects (*p* > 0.05) of *A. armata* exudate exposure or PA-MPs were observed in CAT activity; however, the interaction of these two factors resulted in a significant alteration (*p* < 0.05) in CAT activity (Table S2). Despite that, the *post hoc* tests did not detect statistical differences among treatments (Figure 2a). Considering the GST activity and tGSH levels, no significant effects (*p* > 0.05) of *A. armata* exudate exposure, PA-MPs, and their interaction were observed (Table S2, Figure 2b,c) 

Considering the oxidative damage in the mussels’ digestive gland, no significant alterations (*p* > 0.05) in PC levels were observed when organisms were exposed to *A. armata* exudate, and no interaction of *A. armata* exudate and PA-MPs was observed either (Table S2). However, the PC levels were significantly affected in mussels exposed to PA-MPs (*p* < 0.05, Table S2). The post hoc test revealed significant differences within the 2% exudate concentration (*p* > 0.05) in the levels of PC. A significant increase of PC levels was verified in mussels exposed to 2% exudate in the presence of PA-MPs, when compared to the single exposure of *A. armata* exudate without PA-MPs (Figure 2d). As observed in gills, LPO levels did not exhibit alterations in the digestive gland in none of the treatments (*p* > 0.05, Table S2, Figure 2e). Finally, the exposure to *A. armata* exudate and PA-MPs did not interfere with the activity of AChE, and there was no interaction between the two tested stressors (*p* > 0.05, Table S2).

**Figure 3:**
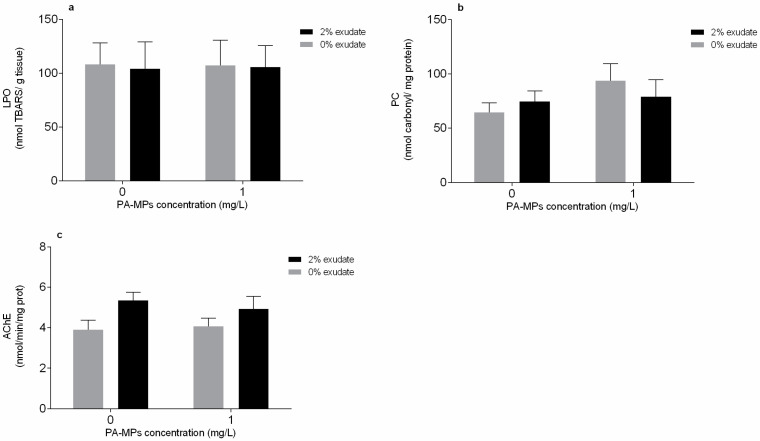
Oxidative stress-related biomarkers of *Mytilus galloprovincialis* muscles after 96 h of exposure to *A. armata* exudate (0% and 2%) at different polyamide microplastic (PA-MPs) concentrations (0 and 1 mg/L). (**a**) Lipid peroxidation (LPO), (**b**) protein carbonylation levels (PC), and (**c**) acetylcholinesterase activity (AChE). All values are presented as mean ± SEM.

In the muscle, LPO (Figure 3a) and PC (Figure 3b) did not undergo significant alterations (*p* > 0.05) when exposed to exudate, PA-MPs, or their interaction (Table S3). On the other hand, the AChE activity was significantly affected in mussels exposed to *A. armata* exudate (*p* < 0.05) but was not influenced (*p* > 0.05) by the presence of PA-MPs or by the interaction between factors (Table S3). Despite that, no statistical differences among treatments were observed (Figure 3c).

### 3.3. Energy Metabolism Biomarkers

**Figure 4:**
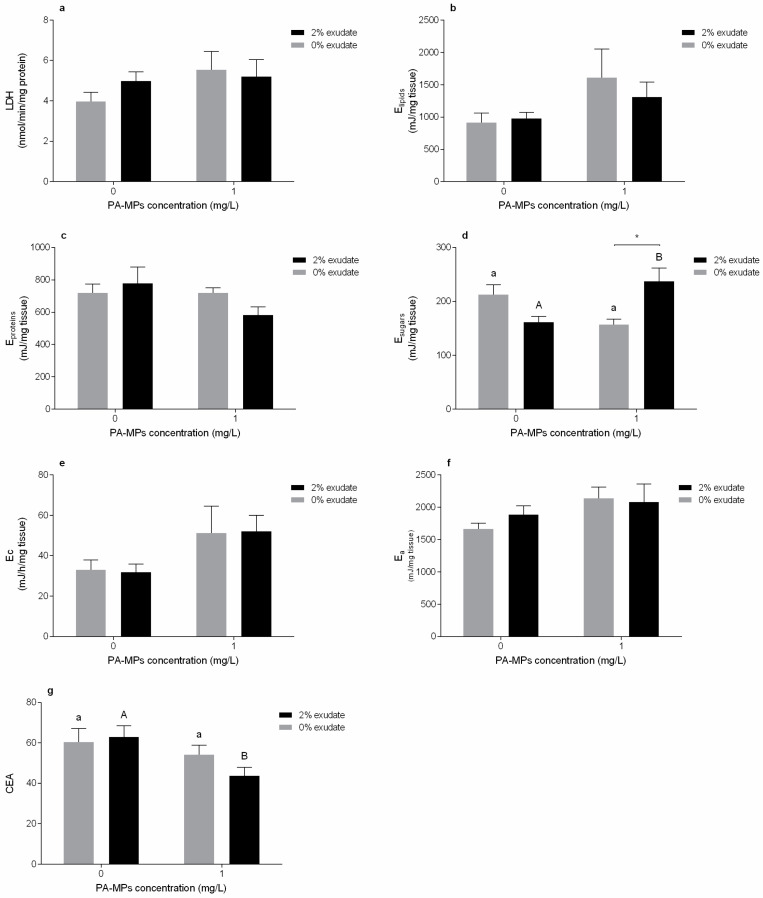
Energy metabolism biomarkers of *Mytilus galloprovincialis* muscles after 96 h of exposure to *A. armata* exudate (0% and 2%) at different polyamide microplastic concentrations (0 and 1 mg/L). (**a**) Lactate dehydrogenase (LDH), (**b**) lipid contents (E_lipids_), (**c**) protein contents (E_proteins_), (**d**) sugar content (E_sugars_), (**e**) electron transport system, (**f**) energy available (Ea), and (**g**) cellular energy allocation (CEA). All values are presented as mean ± SEM. * denotes a significant difference between the 0% and 2% *A. armata* exudate in the same PA-MPs concentration. The upper-case letters indicate differences in the 0% exudate treatments and the different lower-case letters represent differences in the 2% exudate treatments at the different PA-MPs concentrations.

Considering the energy metabolism in the muscle tissue, the activity of LDH (Figure 4a), lipid levels (Figure 4b), and protein content (Figure 4c) were not affected by the presence of *A. armata* exudate or PA-MPs, and there was no interaction between factors (*p* > 0.05, Table S3). In addition, the single exposure to the exudate and the PA-MPs had no significant effect (*p* > 0.05). On the other hand, the interaction between *A. armata* exudate and PA-MPs demonstrated a significant impact on the sugar content (*p* < 0.05, Table S3). There was an increase in sugar content in individuals exposed to PA-MPs in the presence of 2% exudate compared to the single exposure to PA-MPs (Figure 4d, *p* > 0.05). There was also a significant increase of sugar levels in mussels exposed to exudate in the presence of PA-MPs, when compared to the treatment with only *A. armata* exudate (Figure 4d, *p* < 0.05). 

Regarding the aerobic metabolic capacity, ETS activity (E_c_) was impacted in individuals exposed to PA-MPs (*p* < 0.05) but was not affected by the presence of *A. armata* exudate or the interaction of factors (*p* > 0.05, Table S3).These alterations were not reflected in the overall energy available (E_a_) in the presence of *A. armata* exudate (*p* > 0.05), PA-MPs (*p* > 0.05) and there was also no interaction (*p* > 0.05). 

CEA was affected in mussels exposed to the PA-MPs treatment (*p* < 0.05), and there were no modifications in individuals exposed to exudate or both factors (*p* > 0.05, Table S3). There was a significant difference in the 2% exudate concentration (*p* < 0.05), i.e., a decrease in CEA was verified in organisms exposed to *A. armata* exudate in the presence of PA-MPs, when compared to 2% exudate in the absence of PA-MPs.

### 3.4. Byssal Thread Production

**Figure 5:**
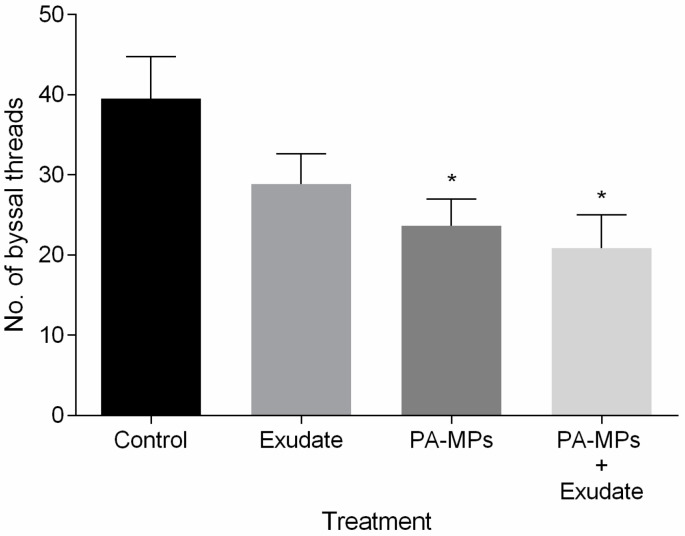
Number of produced byssal threads by *Mytilus galloprovincialis* during the 96 h exposure to different treatments: (i) control (0%; 0 mg/L); (ii) *A. armata* exudate (2%); (iii) PA-MPs (1 mg/L); and (iv) *A. armata* exudate (2%) and PA-MPs (1 mg/L). All values are presented as mean ± SEM. * denotes a significant difference compared with the control treatment.

The number of produced byssal threads was not significantly affected in mussels exposed to *A. armata* exudate (*p* > 0.05). However, a significant decline in the number of byssus was observed in mussels exposed to both PA-MP treatments (with and without the exudate) when compared to control (*p* < 0.05, Figure 5). 

## 4. Discussion

### 4.1. Microplastics in the Tissues

PA-MPs were taken up by *M. galloprovincialis*, as they are mostly found in the digestive gland, which is in line with previous studies exposing bivalves to treatments containing MPs [13,14,70,71,72]. A smaller amount of PA-MPs was detected in the gills. Histological analyses also revealed the presence of few particles retained in the gills epithelium of *M. galloprovincialis* exposed to polystyrene (PS) [73] and to polyethylene (PE) [51], and also of the freshwater bivalve *Corbicula fluminea* [74]. 

The highest number of PA-MP particles was found in the digestive gland under the presence of *A. armata* exudate. This may be explained either by the fact that the exudate presence increased the uptake of PA-MP or the exudate compounds could have compromised the mussels’ ability to excrete these particles. As *A. armata* exudate was shown previously to decrease the clearance rate capacity of exposed mussels [45], the second hypothesis seems to be more plausible. The mechanism underlying this process requires further investigation. In contrast, previous studies investigating the MP effects of co-exposure with other contaminants (e.g. benzo(a)pyrene, fluoranthene) in mussels did not find differences in MP accumulation between organisms treated with MPs alone or in combination [51,73].

### 4.2. Oxidative Stress and Neurophysiological Biomarkers 

Toxicity of MPs and *A. armata* exudate is in part mediated by increased reactive oxygen species (ROS) production, which induces antioxidant defences in the exposed organisms to prevent oxidative damage. Such responses are expected following PA-MP exposure, as this polymer may accumulate in the organisms’ tissues resulting in physical damage, inflammatory responses [13,14], and the consequent activation of defence mechanisms. In addition, *Asparagopsis* seaweeds are a source of halogenated compounds that are inextricably linked to ROS production [75]. Catalase (CAT) is at the first line of defence in the elimination of ROS [76], along with other enzymatic defences, such as superoxide dismutase (SOD). GST has an important role in the phase II of biotransformation and non-enzymatic tGSH acts in the neutralization of ROS [77]. 

In the bivalves, gills have both a respiratory and feeding role and are the first tissue in contact with the stressor [78]. CAT activity in the gills declined in organisms exposed to PA-MPs. H_2_O_2_ is the main precursor of hydroxyl radical in marine organisms [72], and its formation is favoured by ROS production (mainly superoxide anion). CAT may prevent cell damage due to MPs-induced oxidative stress, as this enzyme is involved in the removal of H_2_O_2_ by converting the hydrogen peroxide into H_2_O and O_2_ and acting as a defence mechanism towards exogenous sources of H_2_O_2_ [77]. CAT inhibition was also observed after a 7-day exposure to PS MPs [73]. The authors hypothesised that this enzyme has a biphasic response in the neutralisation of the hydrogen peroxide production, with an activation within the first days of exposure followed by a decrease in activity [73]. Although our study assessed CAT activity after a 96 h exposure, a similar response may also explain the CAT inhibition after this period. Thus, the depletion of CAT activity observed in the PA-MPs treatment may be related with its involvement in the decomposition of hydrogen peroxide. Reduced CAT activity was also demonstrated by Abidli et al. [48] in *M. galloprovincialis* females exposed to PE at 100 and 1000 μg/L. GST activity was not altered in mussels exposed to any of the treatments. Webb et al. [79] also observed no changes in the GST activity in the mussel *Perna canaliculus* gills exposed to 0.5 g PE/L. Furthermore, results suggest a participation of tGSH as second line of defence following the depletion of CAT activity, with mussels from the combined exposure of *A. armata* exudate and PA-MPs presenting the lowest tGSH levels. tGSH is one of the most abundant scavengers in marine organisms that neutralises ROS and acts as a cofactor of various antioxidant enzymes dependent on glutathione [77], and therefore has an important role in the protection against ROS. The decrease in tGSH levels suggests an active involvement in combating excess reactive oxygen species (ROS) by increasing the consumption of total glutathione to counteract a potential increment of oxidative stress caused by the PA-MPs and the macroalga exuded secondary metabolites. Nevertheless, this decline may also reduce the competence for ROS neutralisation, which increases the oxidative damage potential [80]. In fact, although no lipid peroxidation occurred, oxidative damage at the protein level (PC) was observed in mussels exposed to both stressors combined. The imbalance between the generation of ROS and detoxification could have resulted in this rise in protein carbonyl levels. Protein carbonylation (PC) is a type of protein oxidation that can be promoted by the production of ROS [81]. It usually results in the formation of reactive ketone groups or amino acid aldehydes that can lead to the degradation of protein functions [81]. This may increase PC expression in response to different stressors, such as *A. armata* exudate and PA-MPs, thus representing a form of oxidative damage. LPO occurs due to a chain of molecular reactions that can culminate in oxidative damage of lipids allowing toxic agents to penetrate cell membranes [76]. In this study, as LPO was not affected in any tissue, it is not expected that changes in the lipid bilayer’s structure and function or in membrane permeability occurred. Furthermore, the absence of modifications in LPO suggests the efficiency in activation of ROS scavenging mechanisms to prevent oxidative damage at the lipid level [82]. 

Oxidative stress-related biomarkers were also assessed in the digestive gland, which is the main surface for PA-MP uptake after being filtered through the gills, as they are also recognised as an important detoxification organ [83]. CAT activity was inhibited in organisms exposed to the combined exposure to PA-MPs and *A. armata* exudate, and, as in the gills, it is hypothesised that the decrease in this enzymatic antioxidant is due to a strong response in the early stages of exposure leading to its inhibition. Depletion of CAT activity was also observed in the digestive tissue of *M. galloprovincialis* exposed to PE and PS for 7 days [14], *Mytilus* spp. exposed only to PS also for 7 days [73], and the clam *Scrobicularia plana* exposed to 1 mg PS/L for 14 days [72]. On the other hand, GST and tGSH were not altered along the different treatments, which may imply that the second phase of the biotransformation of ROS and detoxification was presumably not activated in the mussels’ digestive glands, at least at the sampling point used. The absence of significant modifications in GST levels in the digestive tissues of mussels exposed to microplastics was previously demonstrated by Avio et al. [14], as well as the unaltered levels of LPO. Cole et al. [45] also did not find significant lipid peroxidation in the digestive gland of *Mytilus* spp. exposed to polyamide microfibers. In response to the PA-MP stress factor, which can trigger inflammation processes in the tissues of exposed organisms [84], there was oxidative damage in the form of protein carbonylation (PC) in the digestive glands of mussels exposed to the polyamide microplastic treatment.

LPO and PC levels remained unaltered in the muscle tissue in mussels exposed to all the treatments, suggesting that no oxidative damage occurred in this tissue. Although antioxidant defence-related biomarkers were not measured, the absence of effects at the protein and lipid levels allows us to infer that the antioxidant machinery was efficient in the muscle tissue.

AChE is generally used to evaluate the neurotoxic potential of various compounds in marine organisms [85] and has an important role in the regulation of cholinergic neurotransmissions [86]. Microplastics-induced neurotoxicity has been previously demonstrated in the mussel *Mytilus galloprovincialis* exposed to 1.5 g/L PE and PS (<100 μm) [14], the clam *Scrobicularia plana* exposed to 1 mg PS/L (20 μm) [72], and *Corbicula fluminea* after exposure to 0.2 mg/L red fluorescent microspheres (1–5 μm) [74]. Therefore, if this enzyme is adversely affected, the essential nervous system functions may be disrupted. However, in the present study, no alterations were detected in the AChE activity of either the gills or the digestive gland, which may indicate that the responses in these tissues were not related to neurotoxicity. On the other hand, the AChE activity exhibited an increase in the muscle tissues of mussels exposed to *A. armata* exudate. Silva et al. [87] discussed that exposure to this seaweed exudate followed by the induction of AChE activity may be related to an induced regulatory overcompensation by increasing AChE in the organisms’ cholinergic system. Another possible explanation is when the AChE is released from the cellular membrane surface, which may trigger *de novo* synthesis to restore this enzyme [88]. Furthermore, this increase in AChE activity may signal an induction of inflammatory reactions, as AChE rise usually occurs in inflamed tissues or cells [89], and may be associated with cell-disrupting processes, especially apoptosis [85]. An AChE activation was previously observed in *G. umbilicalis* [87] and in the muscle tissue of *M. galloprovincialis* [45] exposed to lower concentrations of *A. armata* exudate. However, although previous studies have demonstrated neurotransmission impairment attributed to other MPs, in the present study no effect was observed under PA-MP exposure.

### 4.3. Energy Metabolism Biomarkers

LDH enzyme has an important role in the anaerobic pathway of energy production [90] and was not altered in exposed mussels. Thus, there are no indications of energy mobilisation through anaerobic metabolic vias to counteract stress caused by the metabolites released with the *A. armata* exudate and the presence of PA-MPs.

The energy reserves were measured as lipid, sugar and protein contents, which, in a normal situation, are used in trade-offs between the organisms’ basal maintenance and physiological functions. Lipids and proteins were not altered in neither of the treatments. However, there was a significant increase in sugar levels in organisms exposed to the combined treatment of *A. armata* exudate and PA-MPs. The demand for additional cellular glucose may be related to the induction of gluconeogenesis and may imply a disruption in the energetic metabolism. Lacroix et al. [82] hypothesized that induction of gluconeogenesis could transduce a higher energy storage (in the form of glycogen) in the exposed mussels, but an increased need of glucose to fulfil alternative metabolic routes to combat oxidative stress could also explain this increase. Moreover, the increased gluconeogenesis can be correlated to an increase of reactive oxygen species, as ROS can be generated indirectly by increasing the aerobic metabolism so that organisms are apt to sustain energy costs of metabolic responses to stressful conditions, considering that the electron transport system is a primary site for ROS production [77]. Energy consumption was assessed by determining mitochondrial electron transport system (ETS) activity and may be used to measure the metabolic capacity in response to stress. Mussels exposed to PA-MPs demonstrated increased energy consumption, either with or without the exudate. The increased ETS activity, and consequent increment of aerobic energy production, can be associated with an increase in stress levels while the organisms try to maintain a state of physiological homeostasis [91] and may also support the gluconeogenesis hypothesis. Therefore, this metabolic activation demonstrates a transfer of resources to produce energy, allowing the mussels to cope with microplastics-induced stress. Moreover, a potential increment of non-enzymatic antioxidant capacity is suggested by the ETS increase [45] in the presence of PA-MPs. The increase in energy consumption was accompanied by a depletion of CEA activity in mussels exposed to PA-MPs during 4 days, which ultimately represents a significant decrease in the energy budget; this decline being most noticeable when both stressors are combined. CEA suppression implicates a lower amount of energy available for the mussels’ growth, reproduction, defence, and byssus production, and thus is more susceptible to additional stress [92]. Shang et al. [93] also demonstrated a CEA decline in *Mytilus coruscus* exposed during 14 days to high concentrations (10^4^ and 10^6^ particles/L) of PS microspheres as well as an increased cellular energy demand (ETS activity). On the other hand, Van Cauwenberghe et al. [91] also detected increased ETS activity after exposing *M. edulis* for 14 days to 110 PS microspheres/mL (10, 30 and 90 μm), but this increased metabolism was not accompanied by any other alterations in the overall energy budget.

### 4.4. Byssal Thread Production

Byssus represent an extracellular and collagenous structure that allows mussels’ attachment to the substratum, thus any interference in byssal threads production can diminish the capacity of mussels to firmly anchor to the surface [94], making them prone to dislodgement and more susceptible to natural stressors, such as tides, waves and predation [45]. Production of functional byssus declined in mussels exposed to PA-MPs, either in the presence or absence of the exudate, with a lower number of secreted byssal threads being found under stressor combination. Decreased byssal production was also observed in the mussels *Perna viridis* [71] and *Perna canaliculus* [79] exposed to polyvinyl chloride and polyethylene particles, respectively. 

The exposure to PA-MPs and combined stressors led mussels to allocate more energy to cope with the oxidative stress, which, together with the high levels of protein oxidation, might have compromised the organisms’ ability to invest in the growth and development of structures, such as the byssal threads. Thus, this study suggests that the presence of *A. armata* exudate combined with PA-MPs might increase the vulnerability of *M. galloprovincialis*, as byssal threads are crucial to anchor themselves to the rocky shores and to other mussels. This may consequently impair individuals’ fitness, survival, the preservation of mussel beds, and their role in regulating macrofaunal and flora diversity [78]. 

## 5. Conclusions

In summary, the present findings suggest that 1 mg PA-MP/L in co-exposure with 2% *A. armata* exudate present a health hazard to *M. galloprovincialis*. In particular, the responses of oxidative stress biomarkers and the decrease in the final balance of the energy budget reflected the activation of antioxidant defences in exposed mussels, which prevented lipid peroxidation but not oxidative damage in proteins. Moreover, this was reflected in the impairment of byssus production under exposure to PA-MPs, which can compromise the attachment of mussels to the substratum and mussel bed stability. Thus, a potential amplification of the deleterious effects of the PA-MPs was observed in the presence of this invasive species exudate. This may anticipate that exposure to the secondary metabolites produced by *A. armata* may pose an additional impact to marine biota under the threat of MP pollution.

## Figures and Tables

**Table 1:** Number of polyamide microplastics (PA-MPs) per gram of tissue (gills and digestive gland) in *Mytilus galloprovincialis* exposed to PA-MPs and PA-MPs together with *A. armata* exudate. All values are presented mean ± SEM. ww = wet weight.

Tissue	Number of Particles per Gram Tissue (ww)	
	PA-MP	PA-MP + Exudate
Gills	6.97 ± 3.08	11.95 ± 4.83
Digestive gland	35.04 ± 16.09	62.25 ± 25.98

## Data Availability

The data presented in this study is available in the current manuscript, raw data is available on request from the corresponding author.

## Supplementary Materials

The following are available online at https://www.mdpi.com/article/10.3390/toxics10020043/s1, Figure S1: Irregularly shaped polyamide microplastics (PA-MPs). Images taken at 10× magnification (Zeiss Primo Star light microscope, Jena, Germany), Table S1: Two-way ANOVA analysis results on oxidative stress-related biomarker responses in *Mytilus galloprovincialis* gills with *Asparagopsis armata* exudate and PA-MPs exposures as factors, Table S2: Results for two-way ANOVA analysis on biochemical biomarkers responses in the digestive gland of *M. galloprovincialis* with *A. armata* exudate exposure and water temperature as factors, Table S3: Results for two-way ANOVA analysis on biochemical biomarkers responses in the muscle of *M. galloprovincialis* with *A. armata* exudate exposure and water temperature as factors.
