# Supplementary material for: Co-Exposure with an Invasive Seaweed Exudate Increases Toxicity of Polyamide Microplastics in the Marine Mussel Mytilus galloprovincialis
Source: Toxics. 2022 Jan 18;10(2):43. doi: 10.3390/toxics10020043 (PMC8878234; doi:10.3390/toxics10020043)
Supplement: Supplementary file 1 [file toxics-10-00043-s001.zip › toxics-1533525 proof resubmitted supplementary.pdf]

toxics

Supplementary Materials: Co-Exposure with an Invasive Seaweed Exudate Increases Toxicity of Polyamide Microplastics in the Marine Mussel *Mytilus galloprovincialis*

Filipa G. Rodrigues, Hugo C. Vieira, Diana Campos, Sílvia F.S. Pires, Andreia C.M. Rodrigues, Ana L.P. Silva, Amadeu M.V.M. Soares, Jacinta M.M. Oliveira and Maria D. Bordalo

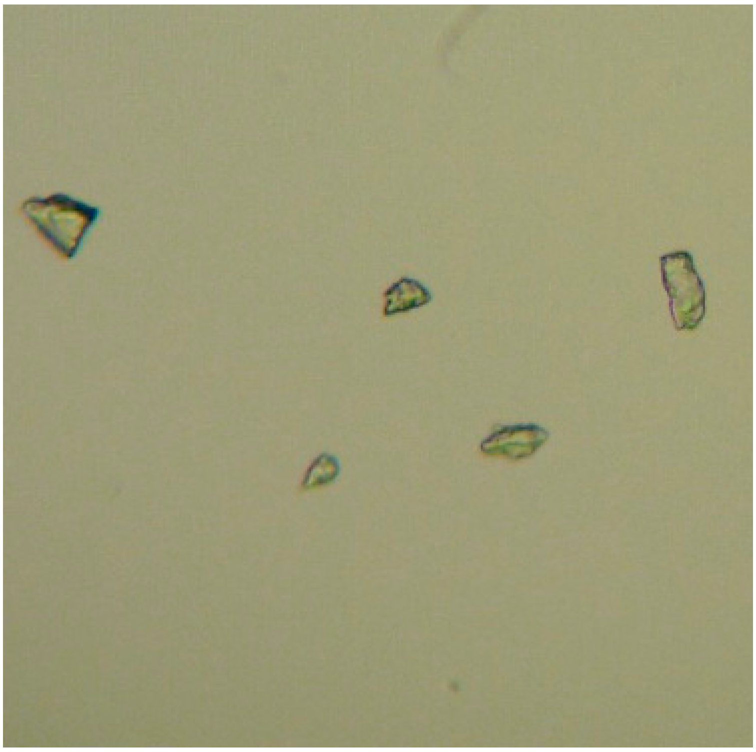

Figure S1. Irregularly shaped polyamide microplastics (PA-MPs). Images taken at 10x magnification (Zeiss Primo Star light microscope, Jena, Germany).

Table S1. Two-way ANOVA analysis results on oxidative stress-related biomarker responses in *Mytilus galloprovincialis* gills with *Asparagopsis armata* exudate and PA-MPs exposures as factors.

Biomarker	Factor	df	F	*p*	% of Total Variation
	Exudate	1, 24	1.594	0.219	4.993
CAT	PA-MPs	1, 24	5.839	**0.024**	18.300
	Interaction	1, 24	0.481	0.495	1.506
	Exudate	1, 24	0.658	0.425	2.669
GST	PA-MPs	1, 24	0.007	0.935	0.028
	Interaction	1, 24	0.001	0.935	0.006
	Exudate	1, 24	0.001	0.979	0.002
tGSH	PA-MPs	1, 24	6.688	**0.016**	18.910
	Interaction	1, 24	4.677	**0.041**	13.230
	Exudate	1, 24	3.038	0.094	8.943
PC	PA-MPs	1, 24	2.558	0.123	7.528
	Interaction	1, 24	4.380	**0.047**	12.890
LPO	Exudate	1, 24	0.261	0.614	0.967

	PA-MPs	1, 24	0.010	0.923	0.035
	Interaction	1, 24	2.707	0.113	10.030
	Exudate	1, 24	0.007	0.932	0.029
AChE	PA-MPs	1, 24	<0.001	0.993	<0.001
	Interaction	1, 24	1.997	0.170	7.680

Bold denotes statistical significance ($p < 0.05$).

Table S2. Results for two-way ANOVA analysis on biochemical biomarkers responses in the digestive gland of *M. galloprovincialis* with *A. armata* exudate exposure and water temperature as factors.

Biomarker	Factor	df	F	*p*	% of Total Variation
CAT	Exudate	1, 24	0.095	0.761	0.331
	PA-MPs	1, 24	0.007	0.935	0.024
	Interaction	1, 24	4.475	**0.045**	15.660
GST	Exudate	1, 24	0.601	0.446	2.399
	PA-MPs	1, 24	0.001	0.978	0.003
	Interaction	1, 24	1.507	0.232	6.017
tGSH	Exudate	1, 24	0.268	0.598	1.023
	PA-MPs	1, 24	3.368	0.079	12.040
	Interaction	1, 24	0.306	0.585	1.094
PC	Exudate	1, 24	2.451	0.131	6.403
	PA-MPs	1, 24	11.814	**0.002**	30.870
	Interaction	1, 24	0.008	0.929	0.021
LPO	Exudate	1, 24	1.359	0.255	5.206
	PA-MPs	1, 24	0.408	0.529	1.562
	Interaction	1, 24	0.344	0.563	1.318
AChE	Exudate	1, 24	0.741	0.398	2.963
	PA-MPs	1, 24	<0.001	0.990	<0.001
	Interaction	1, 24	0.270	0.608	1.080

Bold denotes statistical significance ($p < 0.05$).

Table S3. Results for two-way ANOVA analysis on biochemical biomarkers responses in the muscle of *M. galloprovincialis* with *A. armata* exudate exposure and water temperature as factors.

Biomarker	Factor	df	F	*p*	% of Total Variation
LPO	Exudate	1, 24	0.016	0.899	0.068
	PA-MPs	1, 24	<0.001	0.984	0.002
	Interaction	1, 24	0.004	0.951	0.016
PC	Exudate	1, 24	0.032	0.861	0.118
	PA-MPs	1, 24	1.702	0.201	6.388
	Interaction	1, 24	0.913	0.349	3.426
AChE	Exudate	1, 24	5.552	**0.027**	18.420
	PA-MPs	1, 24	0.065	0.801	0.216
	Interaction	1, 24	0.526	0.475	1.745
LDH	Exudate	1, 24	0.281	0.601	1.044
	PA-MPs	1, 24	1.346	0.257	5.005
	Interaction	1, 24	1.267	0.271	4.711
E_{lipids}	Exudate	1, 24	0.004	0.948	0.015
	PA-MPs	1, 24	4.117	0.054	14.470
	Interaction	1, 24	0.329	0.572	1.156
$E_{proteins}$	Exudate	1, 24	0.347	0.561	1.200
	PA-MPs	1, 24	2.326	0.140	8.037
	Interaction	1, 24	2.270	0.145	7.844
E_{sugars}	Exudate	1, 24	0.707	0.409	1.834
	PA-MPs	1, 24	0.342	0.564	0.887
	Interaction	1, 24	14.499	**0.001**	37.600
E_a	Exudate	1, 24	0.126	0.726	0.456
	PA-MPs	1, 24	2.870	0.103	10.420
	Interaction	1, 24	0.540	0.470	1.960
ETS	Exudate	1, 24	0.066	0.800	0.217
	PA-MPs	1, 24	6.162	**0.020**	20.300
	Interaction	1, 24	0.128	0.723	0.423
CEA	Exudate	1, 24	0.511	0.482	1.577
	PA-MPs	1, 24	6.219	**0.020**	19.190
	Interaction	1, 24	1.679	0.207	5.179

Bold denotes statistical significance ($p < 0.05$).
